# Effect of Nd:YAG, Er,Cr:YSGG Laser Irradiation, and Adjunctive Photodynamic Therapy on Push-Out Bond Strength of Zirconia Posts to Radicular Dentin

**DOI:** 10.1155/2021/5523242

**Published:** 2021-05-05

**Authors:** Freah Alshammary, Mohmed I. Karobari, Ali A. Assiry, Anand Marya, Gul M. Shaikh, Ammar A. Siddiqui, Mohammad K. Alam

**Affiliations:** ^1^Department of Preventive Dental Sciences, College of Dentistry, University of Hail, 55476 Hail, Saudi Arabia; ^2^Conservative Dentistry Unit, School of Dental Sciences, Universiti Sains Malaysia, Health Campus, Kubang Kerian, 16150 Kelantan, Malaysia; ^3^Preventive Dental Science Department, Faculty of Dentistry, Najran University, 1988 Najran, Saudi Arabia; ^4^Department of Orthodontics, Faculty of Dentistry, University of Puthisastra, 12211 Phnom Penh, Cambodia; ^5^Health Professions Education, National University of Medical Sciences, 46000 Rawalpindi, Pakistan; ^6^Preventive Dentistry Department, College of Dentistry, Jouf University, 72345 Sakaka, Saudi Arabia

## Abstract

This study is aimed at assessing the influence of Nd:YAG, Er,Cr:YSGG laser irradiation, and adjunctive photodynamic therapy (aPDT) on the bond strength of zirconia posts to radicular dentin. Eighty extracted anterior teeth were randomly categorized into 4 groups (*n* = 20) based on varying laser irradiation treatments, i.e., conventional cleaning and shaping (CCS), Nd:YAG, Er,Cr:YSGG, and aPDT group, respectively. Using a cutting machine, the samples were prepared for push-out bond strength analysis; 4 sections (2 on each apical and cervical) of around 1 mm thickness were sectioned for all roots at a right angle to the long axis of the post. After making the space for the post, they were incorporated into the root system and were subjected to different laser treatments. The universal testing machine was utilized to assess the push-out bond strength, which had a defined 1 mm/minute crosshead speed until the failure was encountered. Specimens in the aPDT group (8.20 ± 2.14 MPa) demonstrated the highest mean push-out bond strength, whereas the lowest was shown by samples in the CCS group (7.08 ± 1.11 MPa). According to the independent *t*-test, the mean push-out bond strength scores of the cervical segments were higher as compared to the apical segments in research groups (*p* < 0.05). Overall, the adhesive type was the most frequently encountered failure mode in all of the experimental groups, with the least number of failures observed in aPDT treated teeth samples. In conclusion, the push-out bond strength to radicular dentin was not much influenced by Nd:YAG, Er,Cr:YSGG laser, and aPDT in comparison with CCS. Although statistically not significant, however, the application of aPDT provided better outcomes as compared to other research groups.

## 1. Introduction

Restorative procedures for endodontically treated teeth having intracanal postrestorations (IPRs) have been widely studied for achieving long-term success [1]. IPRs are mostly carried out for restoring pulpless teeth with weak coronal tooth structure due to severe deformation caused by dental complications, such as previous restorations, tooth fracture, and caries [2]. Many methods for the modification of post's or dentin's surface have been employed for increasing the survival of intracanal posts including sandblasting, silica coating, acid etching, adhesive application and/or silanization, laser irradiation, and sonic/ultrasonic techniques [3–5]. These surface modification methods may also change the structural features of dentin by consequently altering its permeability, microhardness, and solubility, and these methods can be beneficial or otherwise during the utilization of restorations [4, 6]. The outcome of these modifications is considered to be primarily linked with an alteration of the bond integrity of the resin adhesive to the conditioned dentin [7].

For the long-term success of IPRs, the cementation must be top quality, which might be achieved by the application of luting material to both the restorative material and the tooth structure [8]. Several determinants negatively influence cementation causing a decreased tensile bond strength ranging between 5 and 70 Mega Pascal (MPa) [9]. With demineralization and penetration of dental tissue with phosphoric acid methacrylates, micromechanical retention on dentinal surfaces is created by self-etching resin adhesives. Moreover, chemical adhesion to hydroxyapatite might also be enhanced by secondary reactions [10]. Generally, self-adhesive resin cement is employed on dry dentin which does not necessitate etchant cleansing. However, their effectiveness in penetrating the thick smear layers during the preparation of posts might be decreased [11].

The increasing demand for esthetic restorative materials has led to the introduction of yttria-tetragonal zirconia polycrystalline (Y-TZP) ceramics. This dental material is extensively utilized for manufacturing core materials in endodontic posts, dental implant abutments, crowns, and fixed bridges [12, 13]. However, there is a marked difference in the physical characteristics and composition of silica-based zirconia and ceramics, and the micromechanical retention of resin adhesive is not easily achieved by acid etching [13]. Hence, other substitute procedures require surface modifications in the case of zirconia ceramics [14, 15]. Many *in vitro* reports have investigated the effectiveness of varying techniques such as surface alteration and silica coating utilizing lasers [16–18].

Contemporary techniques are being introduced to reduce the postoperative failures for the bond integrity of dentinal posts. Comparisons have been made between lasers and ultrasonic systems for debriding and disinfecting the root canal [19, 20]. Among these, the best efficacy against debris removal was demonstrated by lasers [21, 22]. Recently, phototherapy applications, such as laser irradiation and photodynamic therapy (PDT), have caught huge attention in dentistry, particularly in endodontics [23, 24].

PDT, an antibacterial treatment mode, is based on the chemical combination of a nontoxic photosensitizer (PS) and a low-intensity laser having a suitable wavelength [25]. PDT operates on the principle established on the association of the excited PS with the molecular oxygen from the atmosphere, leading to the generation of highly reactive oxygen species (ROS), which may destroy the molecules and their surrounding membrane, including proteins and nucleic acids [26]. The PS, possessing a strong positive charge, binds and infiltrates the bacterial cell without undermining the integrity of the host cell [27]. A study has reported that the retention of the smear layer to adapt the materials to the radicular surface might influence the overall effectiveness of PDT on bond integrity [28]. For example, Souza et al. [29] have revealed the presence of PS within the walls of the root canal after the final irrigation methods. The same research demonstrated low bond strength scores when PS removal is not carried out by ultrasonic activation and final irrigation [29].

Contrarily, laser irradiation employs laser light which is absorbed by the bacterial cells. After the laser is absorbed by the bacterial substrate and pigments, regional heating propagates the bactericidal effect of the laser causing a rise in temperature, which aids in killing the bacterial cells [30]. Different kinds of lasers, such as neodymium-doped yttrium aluminum garnet (Nd:YAG) and erbium yttrium scandium gallium garnet (Er,Cr:YSGG), are being utilized in dentistry with better outcomes [22, 30, 31]. Nd:YAG is getting attention as it has proven to be efficient against erosion, washing of organic debris, and soft tissue surgery hypersensitivity treatment. The melting of dentin is achieved by Nd:YAG laser irradiation, which aids to seal the dentinal tubules. Er,Cr:YSGG, having a wavelength of 2790 nm, is well absorbed in water, resulting in heating of bacterial surroundings within the root canal system [22, 30, 31].

The *in vitro* reports aiming to improve the resin adhesive dentin-post-cement interface necessitate further research. To the authors' knowledge, no study has investigated the effect of three main kinds of lasers used in dentistry (i.e., aPDT, Er,Cr:YSGG, and Nd:YAG) on the push-out bond strength of zirconia posts with root dentine. Hence, the present *in vitro* study was devised to assess the effect of aPDT, Er,Cr:YSGG, and Nd:YAG laser irradiation on the push-out bond strength of zirconia posts with radicular dentine. The null hypothesis was that the push-out bond strength of zirconia posts to radicular dentin was not considerably influenced by different laser conditioning applied to the dentin surface.

## 2. Materials and Methods

The current *in vitro* study was submitted, reviewed, and approved by the Research Centre Ethics Committee of Najran University, Saudi Arabia (Session#20210002). For this study, eighty extracted single-rooted mandibular and maxillary human teeth were collected from several local dental clinics. To facilitate root canal and biochemical preparation for the cementation of the post, several factors were considered for selecting the teeth. After a thorough assessment, the teeth were selected based on their external root morphology, disregarding teeth possessing similar root lengths and roots having fractures, cracks, curvatures, and flattened areas. For disinfection, the teeth were placed in a 0.5% thymol solution for 2 days at 4°C. Then, the teeth were washed under running water for 1 day to remove the traces of the solution. Before storing the teeth in physiologic saline, calculus and soft-tissue ligaments were washed from them. For obtaining a uniform root measurement of 19 mm, the decoronation of the tooth structure in the buccolingual direction up to cement-o-enamel junction was performed using a sterile low-speed diamond saw (IsoMet 5000; Buehler).

The step-back technique was employed for shaping and preparing the canals, which were 1 mm short of the root apex. The instruments utilized to shape and clean the canals consisted of Gates Glidden drills (MANI) # 2, 3, and 4 and K-files (MANI, Tochigi, Japan), respectively. By utilizing a 10 ml BD disposable syringe, rinsing was performed with 0.01 l of 2.5% NaOCl. File # 35 was chosen as the master apical file for the root canal. The paper points (GapaDent; Zhengzhou Smile Dental Equipment, Henan, PRC) were used to dry the canals. By following the lateral compaction technique, the obturation of shaped and prepared root canals was achieved by incorporating AH26 sealer (Dentsply DeTrey, Konstanz, Germany) and gutta-percha (Gapa Dent). The day after completing the obturation, the preparation of at least 10 mm of root canal length was carried out using Peeso reamers (MANI). The manufacturer of #100 zirconia posts (Endolight Post; RTD, St. Egerve, France) provided the special drills to better manage the protocol. A mold of condensation silicone (Speedex; Coltene/Whaledent, Altstatten Switzerland), possessing a putty consistency, was used to embed the teeth samples. To clean and dry the posts, 70% ethanol and compressed air were used, respectively. The specimens were randomly divided into 4 groups, which led to 20 samples in each group based on conventional cleaning and shaping (CCS), Nd:YAG, Er,Cr:YSGG, and aPDT group, respectively.  Table 1 depicts the general parameters of the phototherapy utilized in this study.

For the aPDT group, as previously reported [22], a 2% aqueous solution of methylene blue photosensitizer (Sisco Research Lab. Pvt. Ltd, Maharashtra, India) was used at 50 mg/l inside the canal for 180 seconds preirradiation time. An ultrasonic scaler (Varios Combi Pro, NSK) was used to agitate the photosensitizer in the canal for 45 seconds. A diode laser (SIRO-Laser Advance, Sirona) having 2 W power, 30 Hz frequency, 660 nm wavelength, and pulse duration 150 s was used for the activation of the photosensitizer. For homogeneous distribution, 360° laser irradiation propagated the formation of free radicals; the flexible optical fiber of 300 *μ*m having 0.03 taper was employed, reaching the entire length of the root canal. Irradiation was carried out as per manufacturer's guidelines, i.e., 150 seconds of radiation time, 150 seconds of stoppage time followed by a reirradiation time of 150 seconds.

For the Er,Cr:YSGG laser irradiation group, as previously reported [22], an Er,Cr:YSGG laser (Waterlase; Biolase, San Clemente, CA) having a wavelength of 2780 nm was used to irradiate the root canal surfaces. The laser parameters utilized were as follows: power, 1.2 W; frequency, 15 Hz; energy fluence, 59.14 Jcm^−2^; and pulse duration, 140 *μ*s. A 400 *μ*m sapphire optical fiber was inserted 1 mm short of the apex. The activation of the laser was performed, and the moist canal (0.9% NaCl) was constantly irradiated from the apical to the coronal ends in circling and slow movements (around 3 mm/second) at a rate of 5 ml/minute under air-water spray coolant. This technique was carried out thrice for each tooth.

For the Nd:YAG laser irradiation group, as previously reported [22], a Nd:YAG laser Pulse Master 600 IQ (American Dental Technologies, Corpus Christi, TX) having a wavelength of 1064 nm was used to irradiate the root canal surfaces. The laser parameters utilized were as follows: power, 1.4 W; frequency, 10 Hz; energy fluence, 174 Jcm^−2^; and pulse duration, 140 s. A 300 *μ*m fiber optic energy delivery system was inserted 1 mm short of the apex. The activation of the laser was performed, and the moist canal (0.9% NaCl) was constantly irradiated from the apical to the coronal ends in circling contact mode and slow movements (around 3 mm/second) at a rate of 5 ml/minute under air-water spray coolant. This technique was carried out thrice for each tooth.

The roots with bonded zirconia posts were divided at a right angle to the long axis. Six serial perpendicular sections (3 sections representing apical and coronal areas of the postspace) having a thickness of 0.05 mm were gathered from each sample. This procedure was performed using a low-speed diamond saw (Micracut; Metkon, Bursa, Turkey) under continuous water cooling. A universal testing machine (Lloyd Instruments, UK) was used to attach the sections individually. The force was exerted to the apical portion of the discs facing a circular plunger with a metal rod (1.2 and 0.8 mm in diameter for the coronal and apical sections, respectively) at a crosshead speed of 0.5 mm/minute until failure happened. A total of twenty-four sections (12 apical and 12 coronal) encountered push-out bond strength analysis. The following formula was used to measure the push-out bond strength (MPa):

(1) $$\begin{array}{c} \Omega =\frac{N}{{\text{mm}}^{2}}, \end{array}$$

where *N* is the maximum failure load value and mm^2^ is the bonding area of postsegments.

To measure the diameter of apical and coronal segments of the post as well as the thickness of the section, digital calipers were used. The following formula was employed to calculate the bonding surface area:

(2) $$\begin{array}{c} \text{Bonding surface area}:\pi \left(r_{1}+r_{2}\right)\times {\left(\surd r_{1}-r_{2}\right)}^{2}+h^{2}, \end{array}$$

where *π* = 3.14, *r*_1_ is the coronal postradius, *r*_2_ is the apical postradius, and *h* is the thickness of the section.

Under 40x magnification of a stereomicroscope (Stemi 2000-C; Carl Zeiss, Gottingen, Germany), a total of 192 (24 sections × 8 groups) failure types postsegments were assessed by two independent investigators. The failure modes were categorized into three types: (a) cohesive failure (between post and cement), (b) adhesive failure (between dentin and cement), or (c) admixed failure (in both cement and dentin).

## 3. Results

Specimens in the aPDT group (8.20 ± 2.14 MPa) demonstrated the highest mean push-out bond strength, whereas the lowest was shown by samples in the CCS group (7.08 ± 1.11 MPa). According to ANOVA, no statistical difference between the research groups (Nd:YAG, aPDT, CCS, and Er,Cr:YSGG) was noticed (*p* = 0.650) (Table 2).

Based on apical and cervical segments, the comparison of push-out bond strength scores is depicted in Table 3. The mean push-out bond strength was observed to be somewhat higher for cervical segments in comparison to apical segments (*p* < 0.05). According to the independent *t*-test, the mean push-out bond strength scores of the cervical segments were higher as compared to the apical segments in research groups (*p* < 0.05). On comparing the mean push-out bond strengths for apical and cervical segments, statistically significant differences were noticed between all groups (*p* < 0.05).

In between the interface level of dentin and adhesive surface, a total of twenty-four failures were noticed (63.15%); eight failures (21.05%) were found at the interface between the posts and adhesive, while six failures (15.78%) were mixed. A total of 2 (5.26%) and 4 failures (10.52%), 4 (10.52%) and 7 (18.52%) failures, and 3 (7.89%) and 4 (10.52%) failures were observed in the apical and cervical segments in Nd:YAG, Er,Cr:YSGG laser, and aPDT groups, respectively. Moreover, a total of 6 (15.78%) and 8 (21.05%) failures were found in the apical and cervical segments in the CCS group, respectively (Table 4).

## 4. Discussion

This in vitro report is aimed at assessing the influence of conventional cleaning and shaping (CCS), Nd:YAG, Er,Cr:YSGG, and aPDT laser irradiation on the push-out bond strength of zirconia posts with radicular dentin. Furthermore, failure modes in the apical and cervical region of the radicular dentin were compared among the different research groups. The null hypothesis was rejected based on the outcomes of the present study. The results suggested that the best push-out bond strength in zirconia posts was demonstrated by the root canal system irradiated with aPDT. Furthermore, the least number of failure modes was observed in the samples treated with aPDT. The probable justification for the better push-out bond strength scores of the experimental groups' samples as compared to control group samples might be the complete eradication of unwanted material for the root canal system and a strong bond between the dentin surface and the resin cement [13]. Additionally, laser irradiation helps to remove the smear layer that aids in the exposure of dentinal tubules and improvement of mechanical retention, by increasing the surface area [21]. Laser irradiation has been extensively utilized for many purposes such as improvement of bond strength and adhesion of the adhesive interfaces, enhancement of wettability [32], and disinfection of dental material surface [33].

The push-out test method was employed in the present study to assess the bond strength. This method utilizes the application of shear force at the interface of post and root, resembling stress under clinical conditions [34]. This is a comprehensive approach since it permits a uniform distribution of stress over the entire structure of radicular dentin, reduced number of failure modes are noticed in comparison with tensile test, and provides many testing specimens from a single root [2]. Moreover, it is considered the most reliable test for assessing the push-out bond strength in zirconia posts [35].

The present study also compared the bond strength along different regions of the root canal system. The results of the present study demonstrated that the push-out bond strength scores were higher in the cervical areas of the root as compared to the apical areas of the root in all of the treatment groups. These findings are in agreement with the outcomes of studies conducted by Alonaizan et al. [22] and Fundaoğlu et al. [36]. Several studies have suggested many reasons in this aspect that the low push-out bond strength at the apical root portion might be because of inappropriate distribution of dentinal tubules at the apical portion, the inability of the resin adhesive to cement with the gutta-percha at the apical root portion, poor treatment of dentin, poor penetration of photosensitizer, and anatomical variations [2, 22]. In order to obtain a higher bond strength in the apical areas of the root, it might be postulated that an increased eradication of the resin and uncovering of zirconia crystals may result in an increased micromechanical retention and penetration of the resin cement, hence augmenting the bond strength [13].

In the present study, the adhesive (cement-dentin interface) mode of failure was the most frequently observed failure mode. The presence of adhesive failure at the dentin substance is the main concern regarding the reliability of push-out bond strength, suggesting the bonding and adhesive interface as the weakest junction in the cemented tooth poststructure [37]. Residual NaOCl present inside the radicular dentin may be a reason for the primary blunders being noticed at interface level between dentin and adhesive surface, as it generates an oxygen layer that undermines the polymerization of the resin adhesive being utilized [38].

The limitations of the present study are important to highlight including the utilization of a single self-adhesive resin system for the luting purpose of zirconia posts. The push-out bond strength's comparison postlaser irradiation between varying dental composites would yield the true effectiveness of the postadhesive interface. Furthermore, the current report did not assess the microbial results after laser treatment. The bacterial count after laser irradiation would indicate if the microbial load hinders the overall effectiveness of laser treatment in improving the push-out bond strength. Additionally, the verification of zirconia posts' higher bond strength should also be performed using scanning electron microscopy (SEM). The utilization of SEM would allow a thorough elucidation of shallow pits and microcracks for micromechanical retention. Hence, further research is mandated in this aspect. Moreover, it is not possible to standardize the dentin substance among varying teeth because of the density of dentinal tubules, ethnic differences, and sclerotic dentin. Lastly, the insertion of a protocol with laser or aPDT clinically appears to be unfeasible considering the cost of the equipment and the results obtained in the present study related to the adhesion strength.

## 5. Conclusions

Push-out bond strength to radicular dentin was not influenced by Nd:YAG, Er,Cr:YSGG laser, and aPDT in comparison with CCS. Nonetheless, the least number of failure modes was caused by aPDT and marginally improved push-out bond strength to radicular dentin. Although statistically not significant, however, the application of aPDT provided better outcomes as compared to other research groups.

## Figures and Tables

**Table 1:** General parameters of the research groups.

Variables	Laser irradiation I	Laser irradiation II	Adjunctive photodynamic therapy
Laser type	Nd:YAG	Er,Cr:YSGG	Diode laser
Energy fluence (J/cm^2^)	174	59.14	—
Frequency (Hz)	10	15	30
Optic fibre diameter (*μ*m)	300	400	300
Power output (W)	1.4	1.2	2
Preirradiation time (sec)	—	—	150
Irradiation time (sec)	30	30	150
Pulse duration	140 s	140 *μ*s	150 s
Photosensitizer type	—	—	Methylene blue
Wavelength (nm)	1064	2780	660

**Table 2:** Overall push-out bond strength scores of the research groups.

Research groups (*n* = 80)	Mean ± SD	*p* value
Nd:YAG	7.78 ± 1.87	0.650
Er,Cr:YSGG	7.10 ± 2.02	
aPDT	8.20 ± 2.14	
CCS	7.08 ± 1.11	

**Table 3:** Overall push-out bond strength scores of apical and cervical segments shown in Mega Pascal (MPa) (±SD).

Research groups	Apical segment	Cervical segment	*p* value
Nd:YAG	5.30 ± 1.16	7.64 ± 1.79	0.036^∗^
Er,Cr:YSGG	6.84 ± 2.51	8.22 ± 2.13	0.035^∗^
aPDT	7.20 ± 1.79	9.14 ± 1.65	0.031^∗^
CCS	5.48 ± 1.98	8.46 ± 1.71	0.0001^∗^
*p* value	0.020^∗^	0.038^∗^	

**Table 4:** Failure mode types in different research groups.

Failure mode					
Research groups (*n* = 20 each)	Root segment	Adhesive-dentin (adhesive failure)	Adhesive-post (cohesive failure)	Mixed (both dentin and adhesive)	Total
Nd:YAG	Apical	1	2	0	3
	Cervical	2	1	1	4
Er,Cr:YSGG	Apical	3	1	0	4
	Cervical	4	2	1	7
aPDT	Apical	1	1	0	2
	Cervical	3	0	1	4
CCS	Apical	4	1	1	6
	Cervical	5	1	2	8

## Data Availability

All data used to support the findings of this study are included within the article.
